# Scientometric approach to the scientific trends in articles on seagrass in the Atlantic Coast published between 1969-2024

**DOI:** 10.3389/fpls.2024.1484301

**Published:** 2024-12-23

**Authors:** Luiza Reis de Souza, Renato Crespo Pereira, Abílio Soares-Gomes

**Affiliations:** ^1^ Laboratório de Ecologia de Sedimentos, Instituto de Biologia, Departamento de Biologia Marinha, Universidade Federal Fluminense, Niterói, Brazil; ^2^ Programa de Pós-Graduação em Biodiversidade e Biologia Evolutiva, Instituto de Biologia, Universidade Federal do Rio de Janeiro, Rio de Janeiro, Brazil; ^3^ Laboratório de Ecologia Química Marinha, Instituto de Biologia, Departamento de Biologia Marinha, Universidade Federal Fluminense, Niterói, Brazil

**Keywords:** marine botany, marine phanerogam, submerged aquatic vegetation, seagrass ecology, bibliometry

## Abstract

Submerged or partially floating seagrasses in marine or brackish waters form productive seagrass beds, feeding grounds for a rich and varied associated biota, play key ecological roles in mitigating climate change and provide ecosystem services for humanity. The objective of this study was to perform a temporal quali- and quantitative analysis on the scientific production on seagrasses in the Atlantic Ocean during last 64 years (1960 to 2024) through defined workflow by scientometric analysis on Scopus database. Publications in this database date back to 1969, comprising a total of 3.482 scientific articles, primary focused on seagrass ecology. These articles were published in 574 distinct peer-reviewed scientific ecological journals, and are divided into four periods based on the number of articles, keywords and biograms, with an average annual increase of 8.28% in the number of articles published. *Zostera marina*, *Halodule wrightii* and *Thalassia testudinum* were the most researched species, especially in Atlantic coastal areas of Europe and North/Central America. Studies on seagrasses along the Atlantic coast have been well consolidated by a few authors with prolific scientific output, but much of the research has been conducted by non-specialists who published only one or a few articles. We also found that researches from each continent tend to focus on specific topics: North America researches investigated future climate change scenarios and seagrass ecology, while those from Europe prioritize on restoration plans. Additionaly, European researchers from Europe predominantly collaborate with local scientists, a trend also observed among American researches. This indicates a need for increase research and scientific production in the South Atlantic region.

## 1 Introduction

Seagrasses are marine herbaceous phanerogams that can live submerged or partially floating in salt or brackish coastal waters, and they form single-species or multispecies meadows, which provide habitat for various associated biota (Den Hartog, 1970; Copertino et al., 2016). The occurrence of seagrasses as single plants or meadows/patches depends on local hydrodynamic processes (Robbins and Bell, 1994; Fonseca and Bell, 1998). These plants are adapted to thrive in marine and brackish environments, due to several features: (1) linear or sublubated leaves that withstand high-energy environments; (2) hydrophilic pollination; and (3) aerenchyma tissue, which facilitates internal circulation and gas exchange, aiding buoyancy (Hemminga and Duarte, 2001). Seagrasses are typically perennial, meaning their populations persist for more than two years, in contrast to annual or biennial species. Only some populations of *Zostera marina* L., *Halophila decipiens* Asch., and *Halophila tricostata* M. Greenway have been identified as annuals (Kuo and Den Hartog, 2006). However, Lent and Verschuure (1994) observed annual, biennial, and perennial behaviors in *Z. marina* specimens from different parts of the Netherlands, indicating that life cycles can vary depending on habitat conditions. Seagrasses promote species richness and abundance in their habitats by providing food, structure, and refuge, thereby playing a crucial ecological role as foundation species. They create spatial heterogeneity through their branching,canopies and leaves, which serve as biotic substrate for animals and algae (Duarte, 2000; Nakaoka, 2005). Seagrass rhizomes and roots stabilize and oxygenate the sediment, facilitating the establishment of infauna (Duffy, 2006; Copertino et al., 2016). Additionaly, the algae and phytoplankton associated with seagrass meadows contribute to their productivity (Duarte and Chiscano, 1999; Nakaoka, 2005). All these aspects make the seagrasses meadows essential to humankind worldwide due to many ecosystem services (Costanza et al., 1997; Hemminga and Duarte, 2001; Waycott et al., 2009; Duarte et al., 2010; Creed et al., 2023).

Seagrasses act as carbon sinks, with around 40% of their net primary production exceeding the ecosystem’s needs, either being exported or retained in the sediment (Fourqurean et al., 2012). In fact, carbon storage in seagrasses is four times higher than that of macroalgae and they retain approximately 30% of this carbon storage, despite contributing only 4% of marine net primary production (Duarte and Cebrián, 1996). The carbon fixed by marine organisms is referred to as “blue carbon”. Some seagrasses, such as *Posidonia* and *Thalassia* species, have the capacity to store organic carbon in sediments at about twice the average rate of terrestrial soils (Fourqurean et al., 2012).

Despite their multiple ecological roles, seagrass meadows are decreasing and disappearing worldwide, with a loss rate of 27 km² per year between 1879 and 2006 (Waycott et al., 2009). These meadows threatened at the local level by eutrophication and siltation, as well as by large-scale adverse conditions, such as global warming (Orth et al., 2006). If the current rates of seagrass meadow decline continue, it is estimated that 299 Tg of carbon will be released into the atmosphere annualy (Fourqurean et al., 2012). Conversely, Greiner et al. (2013) found that restored seagrass meadows contained three times more carbon and four times more nitrogen in the sediment, enhancing carbon sequestration and helping to mitigate global warming.

From approximately 66 known seagrass species (Den Hartog and Kuo, 2006), 16 are found in the Atlantic coast (see **Table 1**). While some geographic areas, such as the Caribbean region, have yielded fruitful studies on seagrass, others, like the western coast of Africa, have very limited research. In fact, research on seagrass in that region only began around 2018 (Short et al., 2007; Cheikh et al., 2023).

**Table 1:** Seagrass species found in the Atlantic coast, according to Short et al. (2007), along with the biorregions discussed in the present review.

Temperate North Atlantic	Tropical Atlantic	Temperate Southern Oceans
*Cymodocea nodosa+* (Ucria) Asch., *Halodule wrightii+* Asch., *Ruppia maritima* L.*, Z. marina* L., and *Zostera noltii* Hornem,	*Halodule beaudettei* (Hartog) Hartog*, H. emarginata* Hartog*, H. wrightii* Asch.*, Halophila baillonii* Asch.*, H. decipiens* Ostenf.*, H. engelmanni* Asch.*, H. ovalis* (R.Br.) Hook.f*, H. stipulacea* (Forssk.) Asch.*, R. maritima, Syringodium filiforme* Kütz. and *Thalassia testudinum* Meadows & König	*H. ovalis*, *R. maritima, Thalassodendron ciliatum* (Forssk.) Hartog and *Zostera capensis* Setch.

Some of these species are considered invasive in three of the bioregions, and are designated with a “+” (Short et al., 2007).

Due to the significant global variability in the richness and abundance of seagrass species (Short et al., 2007; Fourqurean et al., 2012), it is likely that there are various types of studies on these organisms, as well as differing levels of scientific output. One effective way to assess the scientific production in an area of knowledge is through scientometric analysis. It is derived from bibliometric, that quantitatively examines scientific output and evaluates its progress, identifying overstudies subareas and highlighting knowledge gaps for future research (Mingers and Leydesdorff, 2015). Scientometry allows for the evaluation of numerous scientific studies (Bornmann and Leydesdorff, 2014), through both normative and descriptive approaches (Serenko et al., 2010). Additionaly, these approaches provide a quick and effective way to assess a field of study and enhance our understand of it (Bornmann and Leydesdorff, 2014).

The normative approach establishes standards that guide scientific studies by applying rules based on the Aristotelian logic. Acording to this framework, for a concept to exist, it must be distinct and not overlap with others (Neufeld et al., 2007). In contrast, the descriptive approach employs two methods. The first uses citation counts as a proxy for evaluating the quality of a publication, though it has notable limitations (Meneghini and Packer, 2010). For instance, articles published in languages other than English, those from developing countries, and publications from not-indexed journals are often excluded from citation calculation, which affects many articles from Brazil (Mueller, 1999; Stolerman and Stenius, 2008).

The second method, known as lexical analysis, assumes that scientists use specific words that enhance the thustworthiness of their texts. Analysing these words can yield insights into the strengths and weaknesses of a field of study (Rip and Cortial, 1984; Zitt, 1991). However, lexical analysis also face challenges, such as variations in the publications language and the use of synonyms (Zitt, 1991). Additionaly, this method identifies which knowledge areas are overstudied and which require further research, as well as highlighting the principal authors, leading journals, geographical distribution of studies, publication languages, and publication rates over time (Serenko et al., 2010; Mooghali et al., 2011). It is important to note that the distinction between descriptive and normative approaches can be blurred. For example, a descriptive article that advises on future research directions may inadvertently offer normative recommendations (Serenko et al., 2010).

Although several studies on seagrasses were published worldwide between 1960 and 2015 (e.g. Orth et al., 2006; Short et al., 2007; Fourqurean et al., 2012; Creed et al., 2023), none of them examine how scientific research on seagrasses has progressed along the Atlantic Coast. The present study investigates the scientific production related to seagrass along the Atlantic Coast over the past 55 years using a scientometric approach that addres key research issues and points to future research directions.

## 2 Materials and methods

A broad bibliometric search was conducted in the Scopus database using the following terms: “(seagrass* OR eelgrass OR sav OR amphibolis OR cymodocea OR enhalus OR halophila OR halodule OR posidonia OR phyllospadix OR ruppia OR thalassia OR thalassodendron OR zostera) AND (atlantic OR “Atlantic Ocean” OR usa OR brazil OR spain OR denmark OR uk OR mexico OR Argentina OR “south Africa” OR gabon OR Liberia OR congo OR angola OR gambia OR Mauritania OR sahara OR benin OR gana OR Morocco OR “São tomé and Príncipe” OR “cape verde” OR Namibia OR Senegal OR Camaroes OR guiné OR niger OR “sierra leone” OR “ivory coast” OR congo OR togo OR Canada OR Greenland OR cuba OR Honduras OR Bahamas OR Belize OR Costa Rica OR Guatemala OR Nicaragua OR Panama OR “antigua and barbuda” OR Barbados OR Dominica OR “Dominican republic” OR Grenada OR Haiti OR Jamaica OR “navassa island” OR “puerto rico” OR “saint kitts and nevis” OR “Trinidad and Tobago” OR “Saint Vincent and the Grenadines” OR Argentine OR Colombia OR Guyana OR suriname OR Uruguay OR Venezuela OR Falkland OR Iceland OR Ireland OR France OR Portugal OR Norway OR Belgium OR Netherlands OR Germany OG denmark) AND NOT pacific AND NOT Mediterranean. The search covered the period from 1960 to 2024 (September), as the Scopus does not have information prior to 1960. These terms were searched in the title, abstract, and keywords.

A total of 4.519 articles were found and analysed to exclude those that not specifically focused on seagrasses. This included articles that merely mentioned seagrass as an example of an ecosystem where other organisms occur or studies conducted in regions outside the Atlantic. Articles about experiments with artificial seagrasses and gut analysis were also excluded. However, all other studies on seagrass conducted in the Atlantic Coast, including those in the Baltic, the Wadden Sea, and the Gulf of Mexico, were considered. Although the Gulf of Mexico is described as a ocean in a bowl (see McKinney et al., 2021), it was included in our study because it is geographically part of the Atlantic Coast and host significant seagrass meadows (Thorhaug et al., 2019). Excluding them would render the study incomplete for the Atlantic Coast.

In this study, the Atlantic Coast comprises three bioregions delineated by Short et al. (2006): the North Temperate Atlantic, the Tropical Atlantic, and part of the Southern Temperate Oceans, specifically relating to the coasts of South America and Southern Africa. While the Mediterranean Sea is connected to the Atlantic Ocean, it is classified as a distinct bioregion and was therefore not included in our analysis (Short et al., 2006). Additionnally, articles that were not exclusively about seagrasses but included sampling points in a seagrass meadows were considered as they provide valuable information on important aspects of seagrass meadows in the Atlantic.

Data were analysed using the bibliometrix package in R, specifically through its Biblioshiny application, which transforms coding into a web-based graphical interface (Moral-Muñoz et al., 2020). Biblioshiny supports both Web of Science and Scopus databases, but we chose Scopus because it encompases a broad range of sources (López-Illescas et al., 2008). Additionally, Biblioshiny is recognized as one of the most compreensive scientometrics tools, incorporating features from various other tools (Moral-Muñoz et al., 2020).

Keywords were analyzed based on their centrality (see Cobo et al., 2018; Gandasari et al., 2024 for more details), which measures the degree of interaction with other words, and by density, which assesses the internal strength of the network based on their frequency of occurrence. Using these metrics, the keywords were classified into four categories: motor themes (well-developed and significant to the field), emerging or declining themes (weakly developed and marginal, indicating they are beginning to be studied or ceasing to be studied), niche themes (well-developed internal connections but weak external ties), and basic themes (important for the research field but underdeveloped). Additionaly, a factorial analysis based on Multiple Correspondence Analysis was performed on the keywords.

Clusters of authors were generated based on the frequency of their co-publications. Lotka’s Law analysis (Lotka, 1926) was also performed to determine the authors’s contribution to the advancement of seagrass research in the Atlantic Coast. This law states that the proportion of authors with a certain number of articles, “x”, is a fraction of those who have written only one article, following a power-law distribution of (1/x^a), where “a” is a constant tipically equal to 2. Lotka’s Law also helps to identifing prolific authors within a specific field (Lotka, 1926). Additionaly, we utilised the h-index proposed by Hirsch (2005) to measure the impact of the main journals in the study area. The h-index is determined by the number of publications, “h”, that have received at least “h” citations. These metrics collectively helped quantifing the scientific impact and contribution of authors and journalsin the field.

## 3 Results and discussion

**Figure 1:**
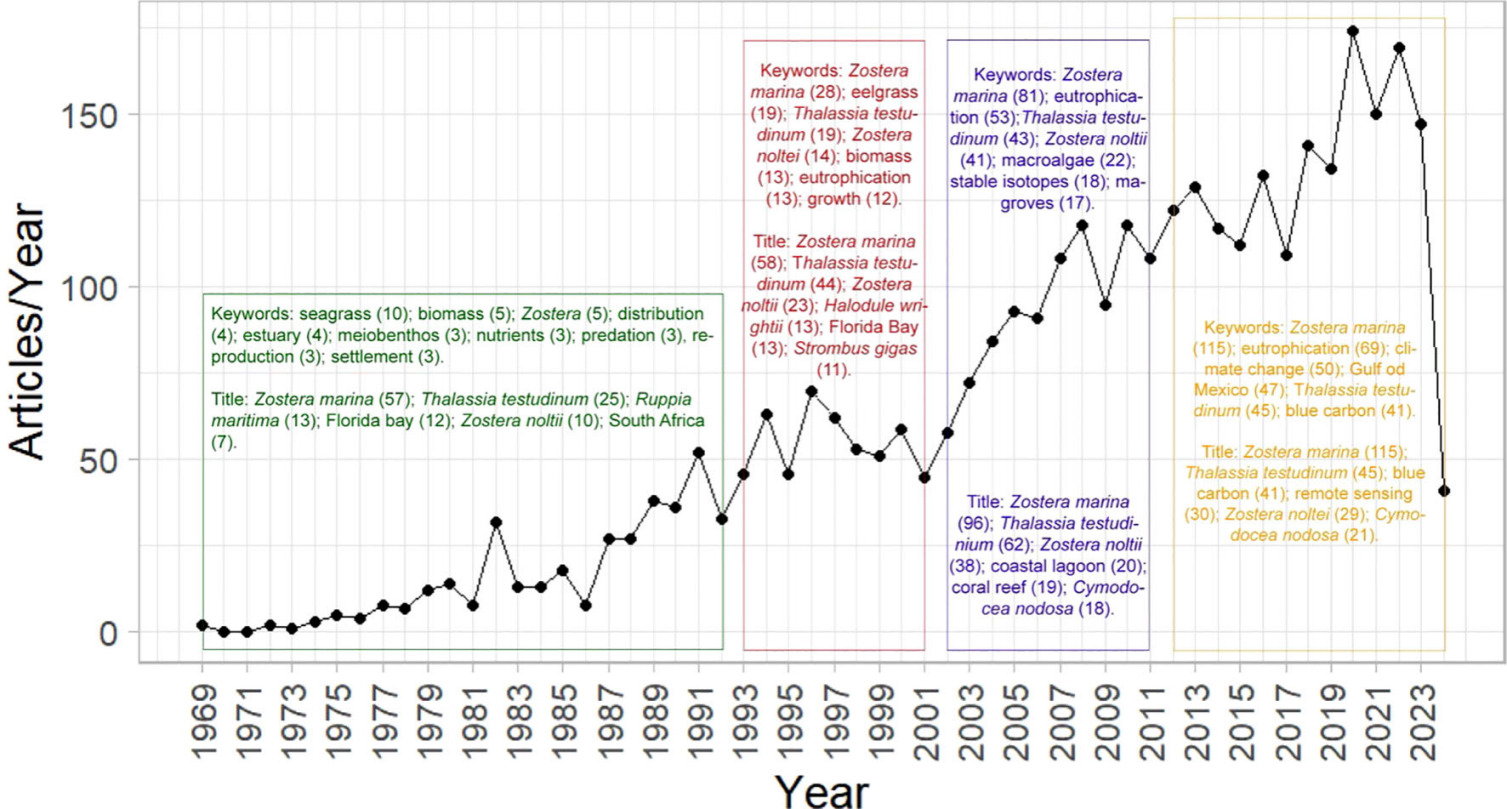
Scientific production on seagrasses in Atlantic Coast during the 1969-2024 timeline is presented, highlighting the most common keywords and title bigrams (combination of two words in the title) for each distinct period: 1969-1992, 1993-2001, 2002-2011, and 2012-2024. The number in brackets following each keyword or bigram indicates the frequency with which it has been cited.

After screening out non-pertinent articles from the 4.519, (**Supplementary Table S1**), a total of 3.482 scientific articles (**Supplementary Table S2**) on seagrass in Atlantic coastal areas were identified, covering the period from 1969 to 2024. These articles are represented qualitatively and quantitatively in a timeline (**Figure 1**), and were published in 574 different scientific journals. This scientific production reflect the diversity of topics covered in seagrass studies in the Atlantic, as highlighted in four phases shown in the timeline. It is also important to note the significance of seagrasses as foundation species; since several articles have also addressed these organisms as habitat, food sources and nursery for a diverse array of fauna.

This timeline is divided into four periods characterized by distinct curve profiles (i.e., number of articles per year), keywords and bigrams cited in article titles that are directly or indirectly related to seagrasses in the Atlantic (**Figure 1**). The first period, from 1969 to 1992, the initial indexing of scientific articles on Atlantic seagrasses (1969) in the Scopus database. This period is characterized by lower scientific production, with a total of 370 articles, compared to later periods (1993-2001, 2002-2011, and 2012-2024), during with the names of seagrass species became more frequent as biograms in the article titles.

It is important to note that studies on seagrasses did not begin in 1969 in the Atlantic Coast, several earlier previous studies date back to the 1930s (e.g. Cottam, 1935), and others expressed concern about the significant decline in eelgrass populations due to pathology (Stevens et al., 1950). However, these earlier studies were published in journals not included in the Scopus database. For example, a chronology on the declining status of eelgrass beds in the Atlantic was conducted in the early 1950s, revealing their absence or scarcity in several locations (Cottam and Munro, 1954).

The trend line from 1972 to 1993 is positive, with slight growth and attention to seagrasses. In fact, studies on the organisms began much earlier, in previous decades not covered by Scopus, such as those in the 1930s (e.g. Cottam, 1934a, b, 1935), the 1940s (e.g. Cottam and Addy, 1947), the 1950s (e.g. Stevens et al., 1950) the 1960s (e.g. Laborel-Deguen, 1963), as well as the 1970s (e.g. Den Hartog, 1970). As recently highlighted, global peer-reviewed publications on seagrasses were rare before 1970, despite the fact that seagrasses exist in most of the world’s coastal zones (Orth and Heck, 2023). According to Orth and Heck (2023), interest in seagrasses began to rise in the 1970s, motivated by the realization of the first “International Seagrass Workshop” held in the Netherlands in 1973, and the publication of significant articles, such as Den Hartog (1970).

The marked increase in the number of articles published since early 1980 can be attributed to several possible aspects. First, the elevated interest in seagrass initiated in the International Decade of Ocean Exploration (1970-1980) during which the Seagrass Ecosystem Study occured, laid a foundational knowledge base. Additionally, the group of 13 participants in this program initiated the first interdisciplinary studies on seagrasses in the USA and launched the first large-scale international collaborations. It took a decade for the graduate students and their mentees to become establish themselves and publish in the seagrass literature, so that by 2000 the stage was set for an explosive growth in seagrass science.

The second period, from 1993 to 2001, was more fruitful than the previous first period (1969 to 1992), with a total of 559 articles (see **Figure 1**). This period not only saw overall increase in the number of articles published but also featured a notable rise in 1996 (71 articles were published) and More species were explored during these time compared to previous period. In addition to *Zostera marina* (34 mentions in keywords and 63 in titles) and *Thalassia testudinum* (22 mentions in keywords and 48 in titles), studies also included *Zostera noltei* (14 mentions in keywords and 24 in titles) and *Halodule wrightii* (15 mentions in titles).

The growth of scientific publications on seagrasses in the Atlantic during the 1990s coincided with a global increase in seagrass research, led by the USA, and marked by an expansion of studies in the North Atlantic (Duarte and Chiscano, 1999). In 1993, the 15^th^ International Botany Congress in Japan hosted the first International Seagrass Biology Workshop, a three-day seagrass workshop where participants focused on global issues related to seagrasses and high-diversity areas (Coles et al., 2014). By 2000, the World Seagrass Association was formed to facilitate international meetings and provide a forum for researchers to share information about seagrasses (Orth and Heck, 2023). Thus, the 1990s and early 2000s were pivotal for the study and conservation of seagrasses, likely contributing to the increase in academic output during this period.

The third period, from 2002 to 2011, is characterized by a variable but high number of articles totaling 1,016 articles (see **Figure 1**). This period continued to explore themes common to the previous period (1992-2001), including *Zostera marina* (84), eutrophication (57), *Zostera noltei* (48), *Thalassia testudinium* (41), macroalgae (35) studies. Among the biograms, we found terms such as *Zostera marina* (98), *Thalassia testudinium* (65), *Zostera noltei* (43), coastal lagoon (22), coral reef (20), *Cymodocea nodosa* (20), Wadden Sea (15), and Baltic Sea (12). Notably, the emergence of published articles on *C. nodosa*, a species from the Canary Islands, may indicate a milestone in the growth of seagrass research in this region. Additionally, the European species *Z. noltei*, also well-studied in the previous period, saw an increase in publications, being mentioned in at least 48 articles.

The fourth and most recent period, from 2012 to 2024, is marked by a total of 1.528 articles (see **Figure 1**). This period continued the high production seen in the previous one (2002-2011), exploring the themes such as *Zostera marina* (103 articles), eutrophication (54), climate change (47), *Thalassia testudinum* (44) the Gulf of Mexico (43) and blue carbon (39). Notably, 176 articles were published in 2020. Regarding bigrams, the most frequently cited terms were *Zostera marina* (118 mentions), highlighting the importance of this species for studies in Europe and North America. This was followed by *Thalassia testidinum* (53), *Zostera noltei* (45), *Cymodocea nodosa* (31) and blue carbon (30), the last indicating a growing interest in climate change.

The scientific journals with the highest number of published articles in this field, in a descending order, were: *Marine Ecology Progress Series* (308 articles), *Aquatic Botany* (212), *Estuarine Coastal and Shelf Science* (175) and *Journal of Experimental Marine Biology and Ecology* (148). Several factors can influence the choice of a journal for publication, including visibility, impact factor, the efficiency of the editorial office and publication costs (Thompson, 2007). Based on our results, it appears that the selection of scientific journals to publish studies on Atlantic seagrasses was largely influenced by these four journals which focus primarily on aquatic ecology, encompassing both marine and estuarine environments.

In our study, the average annual increase in the number of scientific articles published on Atlantic Coast seagrasses (5.69%) was smaller the 19.5% year-on-year growth observed in China (Xiao et al., 2020), which considered articles published since 2010. During the same period (2010-2019), scientific production on Atlantic seagrasses was significantly lower, annual growth rate just 1.41%. The growth in China was likely attributed to an increased funding for research (Xiao et al., 2020), and the hosting of the “International Seagrass Biology Workshop” in China in 2014, which may have positively impacted seagrass studies in this country. Xiao et al. (2020) noted that, overall, USA and Europe still have more seagrass publications, although it is important to mention that not all of these publications pertain to the Atlantic Coast.

**Figure 2:**
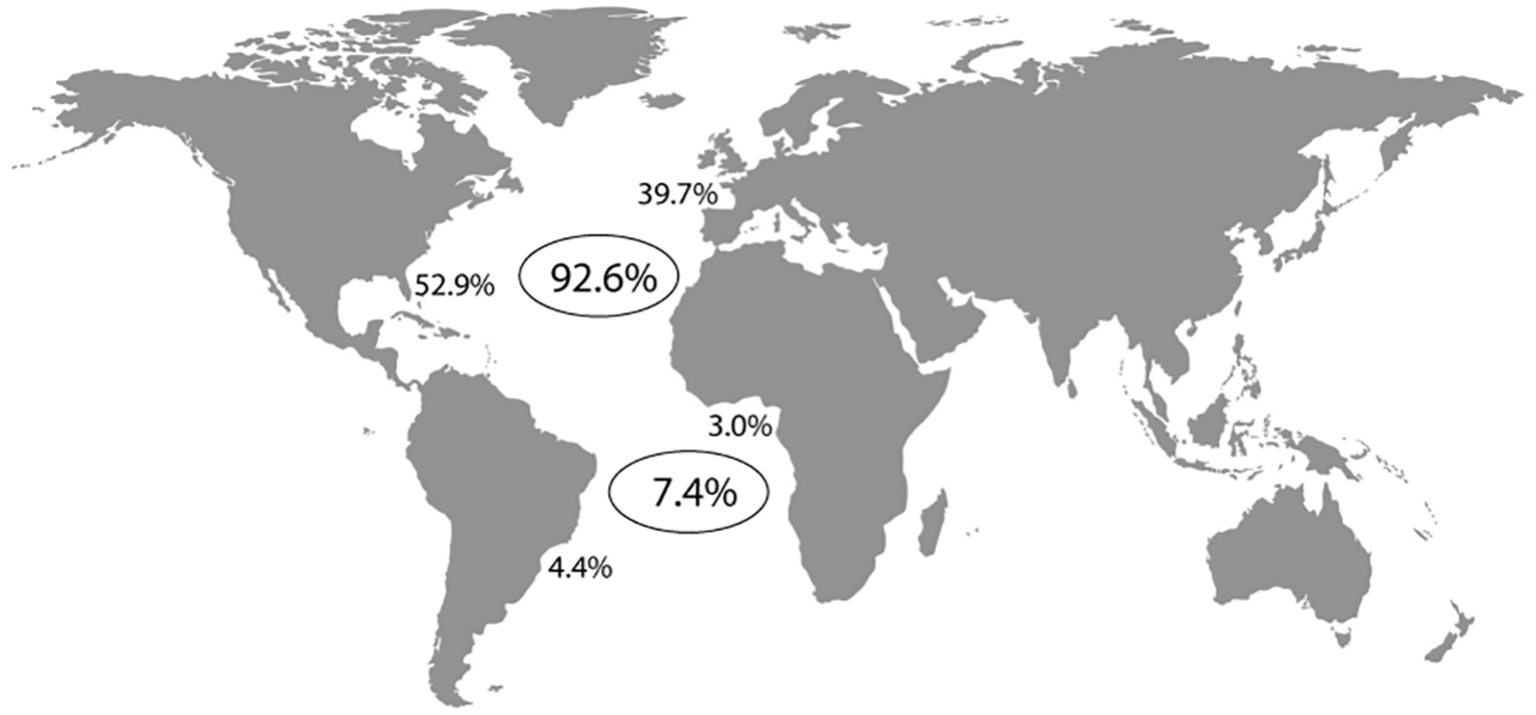
Percentages of total articles published on seagrass studies in the North Atlantic (92.6) and South Atlantic (7.4), as well as in North America (52.9), Europe (39.7), South America (4.4), and Africa (3.0).

A comparison of scientific production on a latitudinal scale revealed that most studies have focused on species and environments in the North Atlantic (92.6%), compared to the South Atlantic region (7.4%) (**Figure 2**).This disparity makes perfect sense, as major scientific institutions are located in the the North Atlantic region. These percentages of seagrass studies stem mainly North America (52.9%) and Europe (39.7%), in the North Atlantic, while studies in the South America (4.4%) and Africa (3.0%) are far less frequent, reflecting a geographic imbalance in seagrass research across Atlantic coastal areas. A recent review identified a total of 46 articles on seagrasses from Africa (Mwikamba et al., 2024), but only six were from the littoral’ West Coast. Of those, four were included in our results (Cheikh et al., 2023; Boutahar et al., 2020; Mvungi and Pillay, 2019; Pillay et al., 2010), while two (Phair et al., 2020; Chefaoui et al., 2021)) were not found in the Scopus search, as that did not use the words “Atlantic”, or the term “Atlantic Ocean” or the name of the country in their title, abstract or keywords. Our study encompassed more research from Africa (424) because it also considered papers focused on biota in seagrass meadows, even if angiosperms were not the primary focus. This indicates that seagrass ecosystems are present along the coast, despite limited study. Notably, nearly all the discussion in our scientometric analysis regarding the scientific development of seagrass studies in the Atlantic are based on information from the North Atlantic.

The limited studies conducted in the South Atlantic clearly indicate a gap in our understanding of various aspects of biology and ecology in this region, which may compromise biogeographical studies for the Atlantic Coast and beyond. Seagrass meadows and submerged aquatic vegetation are present along the entire Brazilian coastline; however, the amount of scientific information is unevenly distributed. Greater knowledge is concentrated in a few areas, notably northeast (Pernambuco state), southeast (Rio de Janeiro state) and south (Rio Grande do Sul state), while other regions remain poorly studied (Copertino et al., 2016). We believe that this uneven profile results from the presence of professionals focused on seagrass research in these 3 Brazilian states, both historically and more recently. This discontinuity is reflected in the apparent absence of certain seagrass species along the Brazilian coast, which may represent a Wallacean shortfall - gap in our understanding of species distributions. Indeed, seagrasses from South America are rarely included or cited in global reviews and models (Copertino et al., 2016). Africa faces an even larger Wallacean shortfall along its West coast, where few studies exist, with most research concentrated on the East Coast (Mwikamba et al., 2024). However, a new project is underway in the West Coast aimed at conserving and mapping seagrasses (Cheikh et al., 2023), which will enhance our knowledge of this neglected region. A recent review emphasized the urgent need for more accurate estimates of seagrass extent in at least 70% of the most relevant African nations, as well as better understanding the factors drives seagrass decline in this area (Mwikamba et al., 2024).

**Figure 3:**
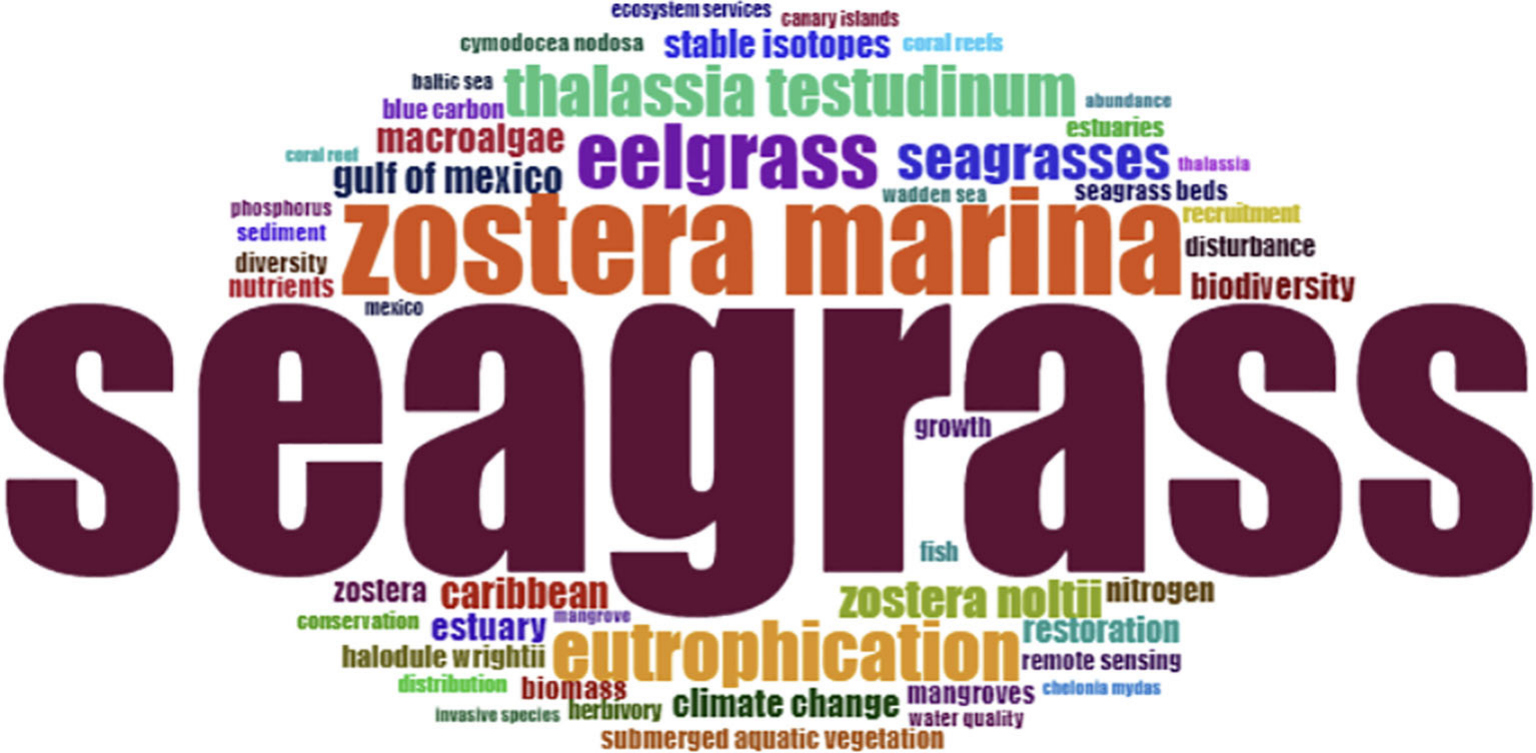
Most frequently used keywords in articles published on Atlantic seagrasses in the period analysed, 1972-2023. The general term ‘seagrass’ was omitted in order to highlight the occurrence of other related terms, such as *Zostera marina*, eelgrass and others.

Among the keywords in published articles from 1969 to 2024, the general term “seagrass” was the most cited (660), followed by *Zostera marina* (224), which occurs in mono-specific meadows throughout most of its global distribution (Moore and Short, 2006). Other frequent keywords included the general term “eelgrass” (138), and to a less extent, *Thalassia testudinum* (108) and *Halodule wrightii* (48) (**Figure 3**). *Thalassia* has more restricted geographic occurrence, primaly comprising coastal regions of the tropical North Atlantic (Strydom et al., 2023). Although less studied, *H. wrightii* is widely distributed in both the temperate and tropical North Atlantic (Caribbean), as well as in temperate and tropical Pacific, and temperate Southern Oceans (Short et al., 2007; Rivera-Guzmán et al., 2017). Another frequently mentioned term is “eutrophication” (125 citations), which is a primary cause of seagrass decline (Burkholder et al., 2007).

**Figure 4:**
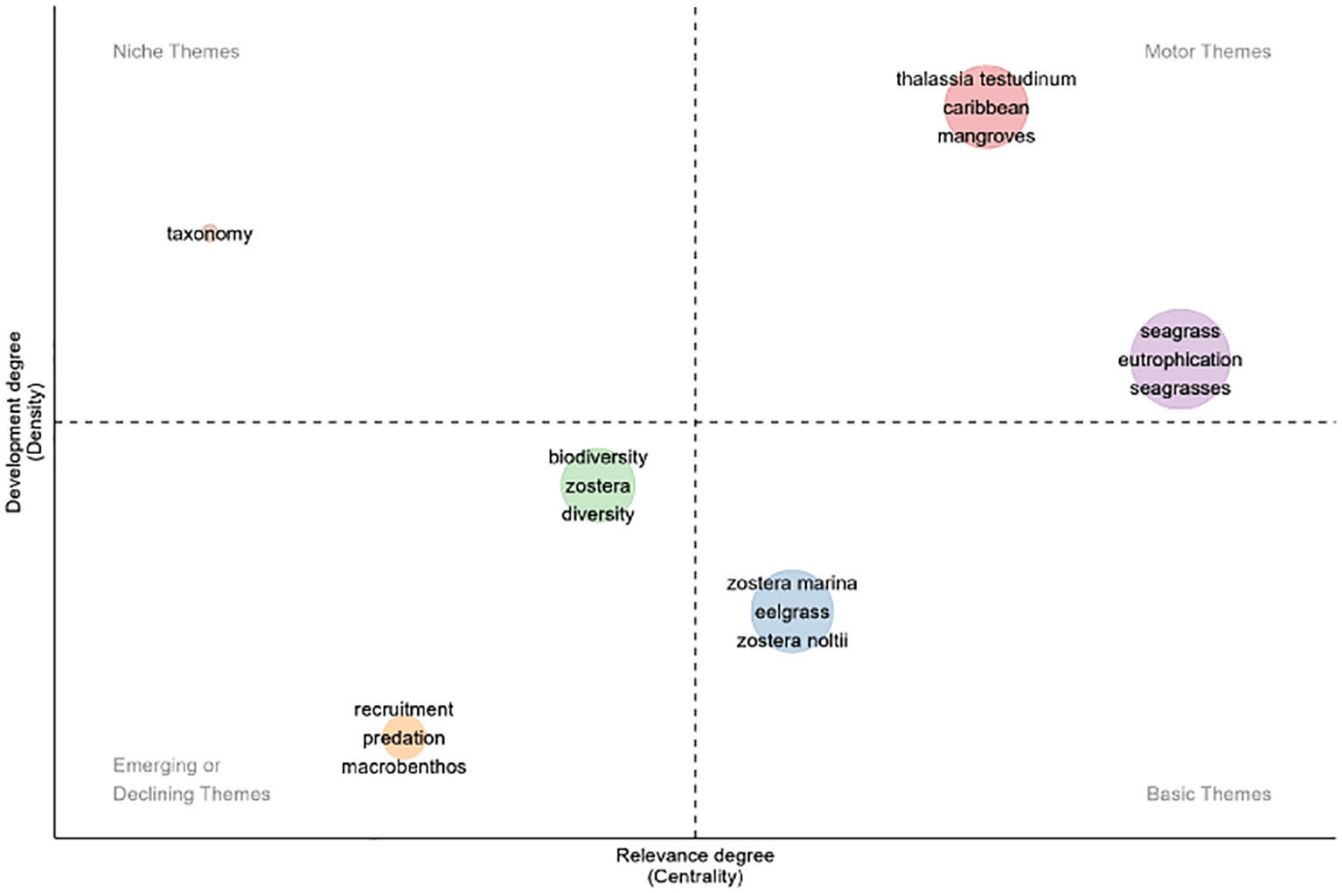
Keywords classified by clusters of occurrences and categorized by their development and relevance into four themes: niche themes, motor themes, emerging or declining themes and basic themes, for the timeline of 1969-2024. The x-axis measures the relevance of the topic for seagrass research, while the y-axis indicates the level of development of the topic.

A growing number of studies have been accompanied by an increasing diversity of topics (**Figure 4**). Research has focused on various ecological interactions, including fish recruitment (e.g. Switzer et al., 2015; Flaherty-Walia et al., 2015; Gorecki et al., 2022), predation of macrobenthos (e.g. Hovel et al., 2021; Siqueira et al., 2021; Gross et al., 2022), and the biodiversity associated with these environments (e.g. Jankowska and Wlodarska-Kowalczuk, 2022; García-Trasviña et al., 2023; Navarro-Mayoral et al., 2023). Several studies also focused on the restoration of *Z. marina*, reflecting a growing concern in Europe today. These emerging themes indicate that while gaining attention in the literature, they remain relatively underdeveloped (Cobo et al., 2018). Studies on taxonomy (e.g. Calder, 2019; Govindarajan et al., 2019; Trott et al., 2020) and ecology (e.g. Armenteros et al., 2021; Chiquillo et al., 2023; Geäfnings et al., 2023) were common in the Caribbean Sea region, categorized as a niche theme due to their specific focus on seagrasses, which have not received much attention from the broader scientific community (Cobo et al., 2018). While these studies are well-developed, they tend to be primarily relevant to seagrass scholars.

Among the motor themes identified in seagrasses studies conducted in the Atlantic Coast, which are considered the most advanced and significant for the field (Cobo et al., 2018), notable research includes work on the Caribbean seas, particularly regarding *T. testidinum* (e.g. Koch et al., 2022; Fourqurean et al., 2023; MacLeod et al., 2023) and the relationship between seagrasses and mangroves (e.g. Thorhaug et al., 2019; Sullivan et al., 2021; Rodemann et al., 2023). Key themes included studies on the effects of eutrophication on seagrass meadows, as well as the ongoing need to understand how to manage these impacts and the decline of these ecosystems (e.g. Burkholder et al., 2007). On the other hand, there are fundamental topics in seagrass research that remain underdeveloped (Cobo et al., 2018), such as studies on the genus *Zostera*, which supports diverse marine life and has impired numerous investigations into its associated biota.

**Figure 5:**
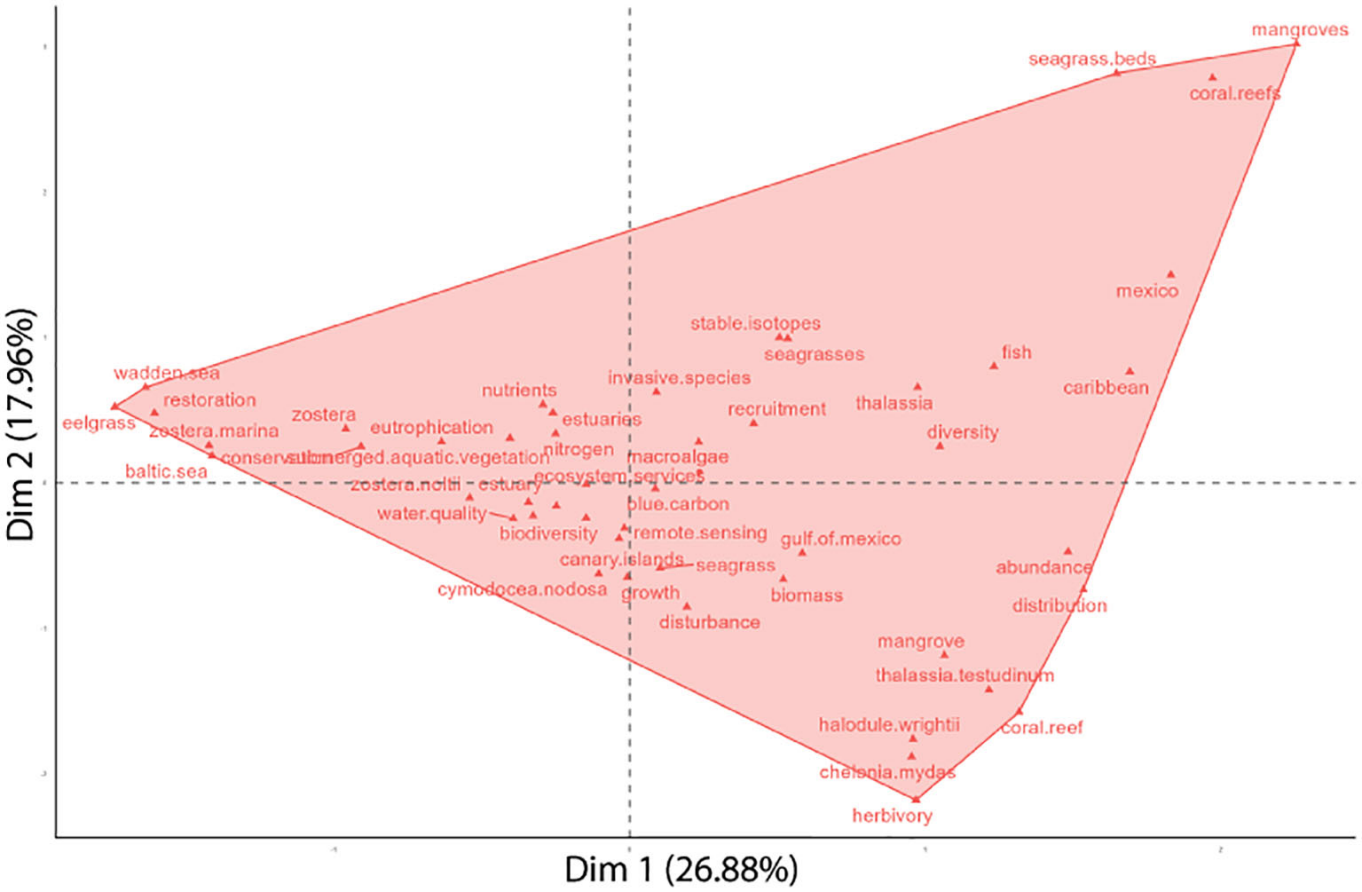
Factorial analysis of the keywords cited in the articles published from 1969-2024.

A factor analysis of the keywords cited in the articles (**Figure 5**) revealed that seagrass species are related to various aspects across the target of the articles. For example, the species *Z. marina* was commonly investigated in studies foused on restoration (e.g. Pastor et al., 2022; Geäfnings et al., 2023; Kwakernaak et al., 2023), particularly in the Baltic and Wadden Sea. On the other hand, *T. testudinum* was primarily studied in the context of herbivory, especially regarding the effects of overgrazing by the green turtle *Chelonia mydas* (Fourqurean et al., 2019; Rodriguez and Heck, 2020) and feeding by the sea-urchin *Lytechinus variegatus* (Roth et al., 2023). Additionally, research exploring the ecological relationships between seagrasses and mangroves, as well as between seagrasses and coral reefs, has been conducted, highlighting their close ecological connections (e.g., Vaslet et al., 2012; Claudino et al., 2015; Ramirez et al., 2017). Studies addressing climate change have predominanrtly focused on *T. testudinum* (Roth et al., 2023) or *H. wrightii* (e.g., Chefaoui et al., 2021).

**Figure 6:**
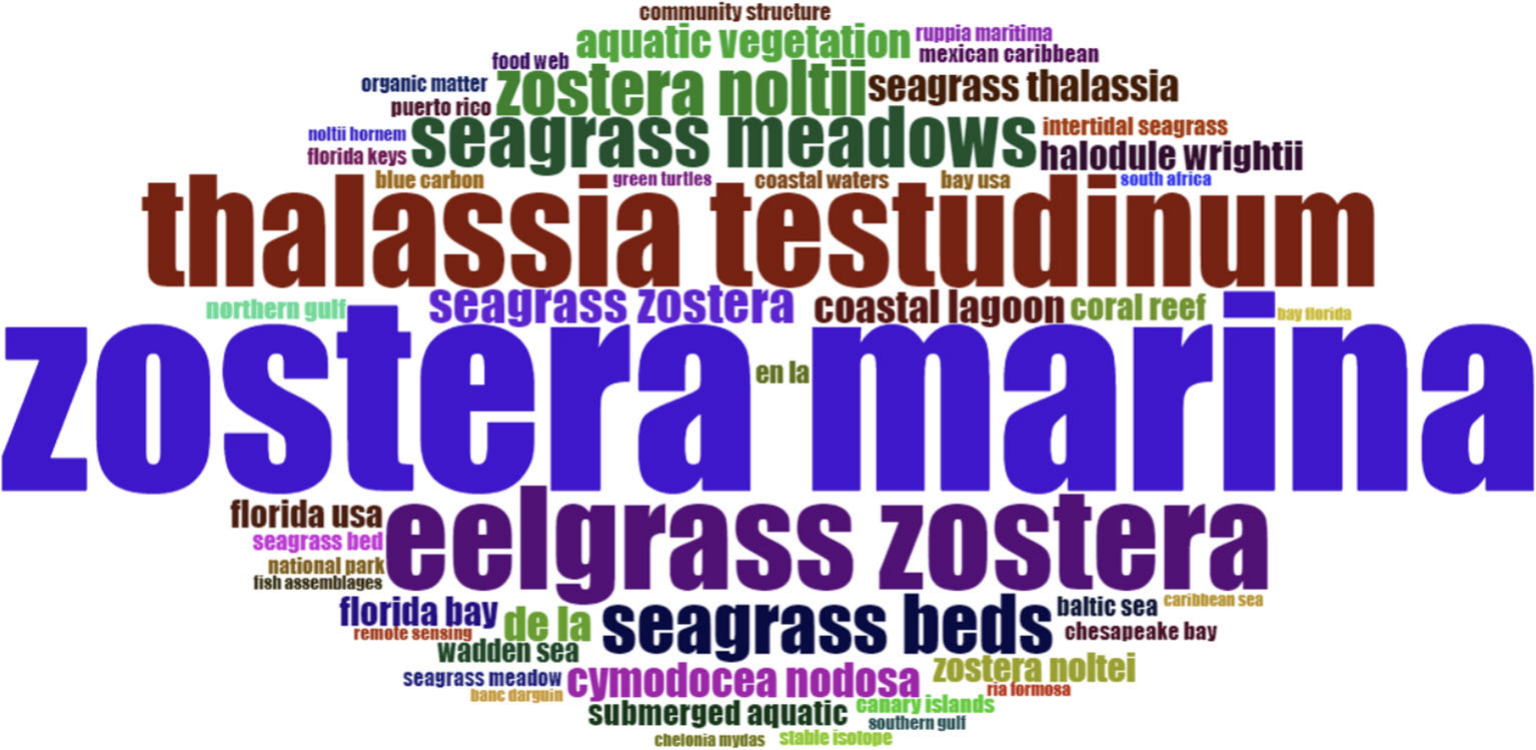
Bigrams (combination of two words) that were most frequently used in the titles of articles published on seagrass in the Atlantic in the period analysed 1969-2024.

Regarding the most cited bigrams (combination of two words), *Zostera marina* (336) emerged as the most frequently cited in the article titles (**Figure 6**). Other notable bigrams include *Thalassia testidinum* (191), eelgrass *Zostera* (177), *Zostera noltii* (94), *Cymodea nodosa* (60), and *Halodule wrightii* (51) The higher citation count for *Thalassia testudinum* is likely due to its status as one of most common, abundant, and widely distributed seagrass species in the Atlantic (Onuf et al., 2003). While keywords are not typically expected to be cited in article titles, this guideline is not always followed, which may explain the observed similarity between keywords and biograms. Another important aspect revealed by biogram analysis is the geographic distribution of seagrasses along the Atlantic, with notable areas including Florida Bay (49), Florida, USA (46), the Canary Islands (31), Chesapeake Bay (31), the Florida Keys (28), South Africa (25), Ria Formosa (24) andBanc DÁrguin (23). Most of these locations are associated with the Gulf of Mexico, although South Africa, Portugal and France are also represented. Finally, stable isotopes analysis (26) was noteworthy, as it is often linked to food web studies (e.g. Dromard et al., 2017; Garcia et al., 2017; Carreón-Palau et al., 2018).

**Figure 7:**
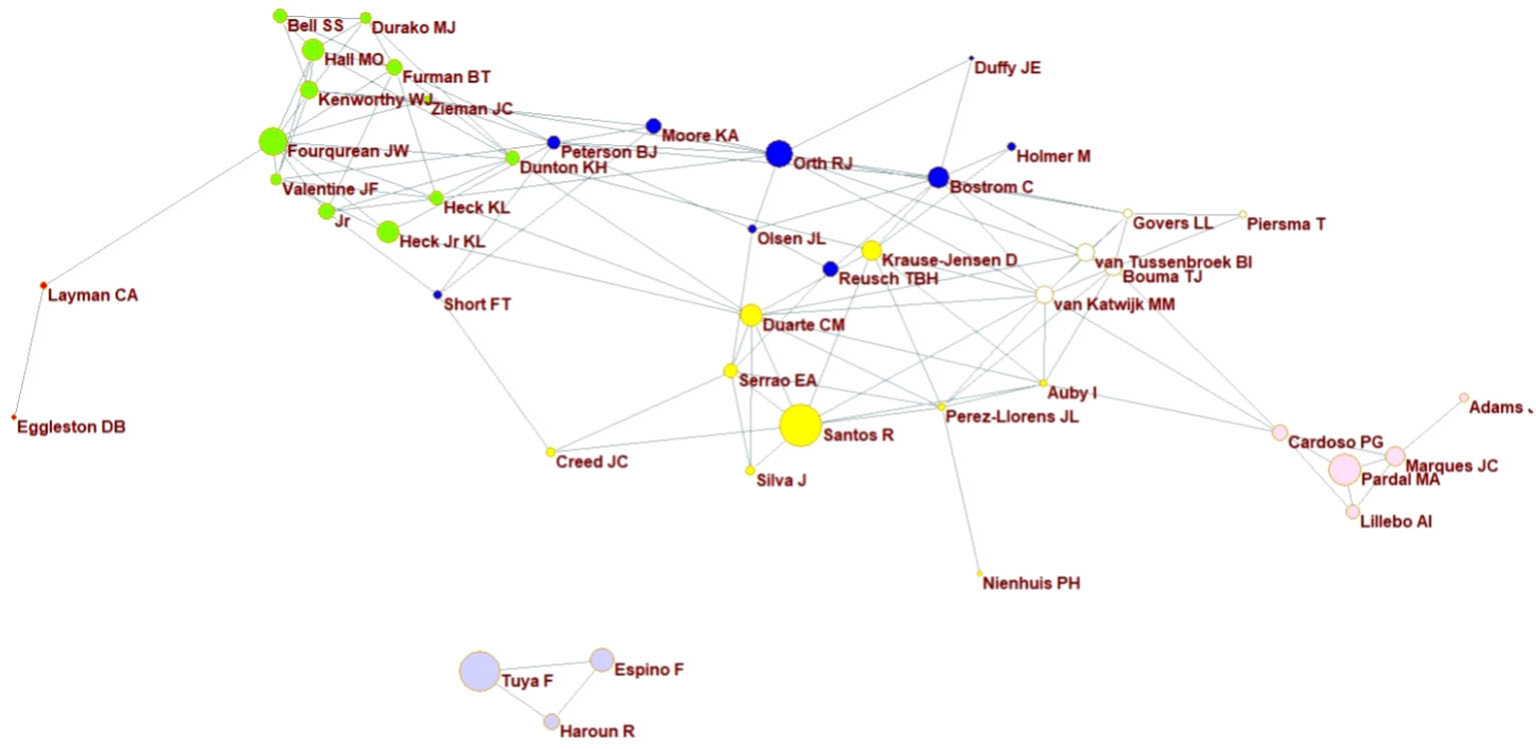
Clusters of authors formed by co-publishing during the analysed period of 1969-2024 are illustrated, with each colored cluster representing the most frequent collaborations. The lines connecting the points indicate additional collaborations both within and between the clusters.

Regarding to the number of articles published by each author, the most prolific were Santos, R. (49 articles), followed by Tuya, F. (41), Orth, R. J. (36), and Fourqurean, J. W. (15) – two European and two American authors. An interesting observation is that Tuya, F., Espino, F., and Haroun, R. form a close cluster (**Figure 7**, in lilac), indicating that they frequently co-published, with a total of 42 articles between them. All of these authors are based in Europe. This finding reiterates the significant discrepancy previously highlighted in the comparison of scientific production on seagrasses between authors from institutions in the North and South Atlantic.

Another interesting aspect of author collaborations (**Figure 7**) is the interaction between different clusters. While authors primarily collaborate with those from their own country or continent such as the pink cluster representing Portuguese authors (and one South African), they also engage in cross-continental collaborations. Recently, there was an improvement in collaborative studies, as indicated by an increase in joint publications. This trend is benefical for science, as sharing knowledge fosters futher advancements (Abbasi and Altmann, 2011). Collaboration between different countries offer significant advantages, overcoming local limitations, and allowing for comparisons across varying conditions (Larsson et al., 2005). It is evident that knowledge from overseas is being shared. However, it is worth noticing that the network of authors from the North Atlantic - comprising professionals, visisting scientists and posdoctors rarely connects with or includes authors from the South Atlantic. Strenghering these connections is crucial for the scientific development of various aspects of seagrasses research.

**Figure 8:**
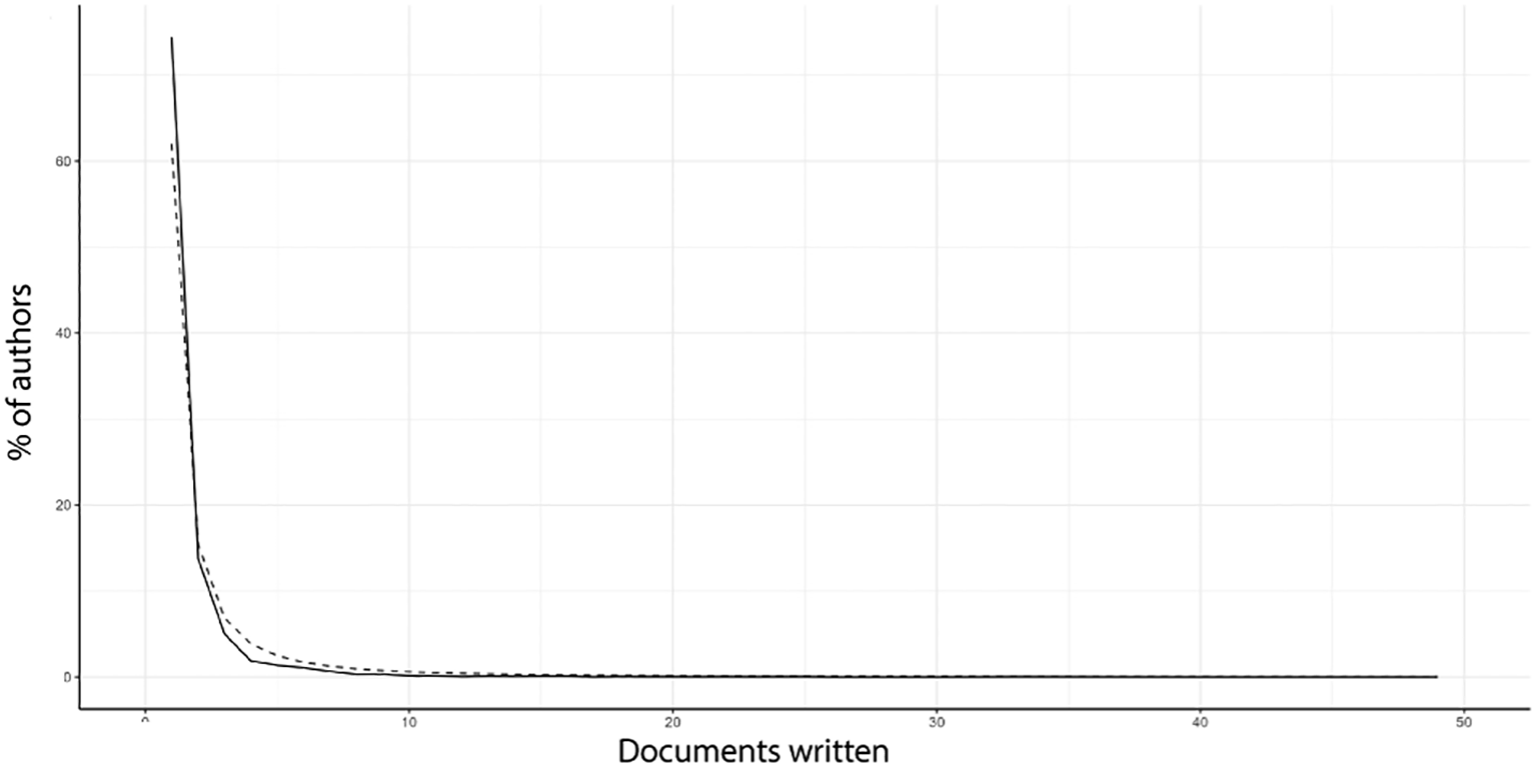
Author productivity analysed under Lotka’s Law reveals that the solid line represents the typical behaviour of Lotka’s Law, while the dashed line reflects our results, indicating that seagrass’ publications and their authors closely adhere to the Lotka’s Law.

The author’ contributions align with Lotka’s Law (**Figure 8**), with 6.393 authors (78.7%) publishing just one article. This finding is ilustrated by the significant proximity and nearly complete overlap between Lotka’s Law (solid line) and articles per author (dashed line). At the opposite extreme, a very low percentage of authors (< 0.001) published 49 articles. This analysis revealed that studies on seagrasses in the Atlantic Coast constitute a well-established and productive scientific area, primarily led by a few prolific researchers. However, most authors are not specialists in seagrasses; they tend to focus more broadly on related fileds, such as coastal environments, and have published only one or a few articles on seagrasses. This finding along insights from the keyword analysis presented earlier, highlights the contribution of non-specialists, indicating that the studies were not strictly or exclusively centered on seagrasses, but rather on coastal environments in which these angiosperms are prominent components.

In the context of authors, the most cited were Heck (27 citations) and Orth (also 27), followed by Fourqurean (26), Kenworthy (24) and Santos (24). Boström and Tuya had the highest h-index (16), followed by Nagelkeren (13). This suggest that there are more articles on seagrass with a higher number of citations among those analysed. A h-index of 13, for example, does not imply that none of these articles have more than 13 citations: in fact, it is likely that some do. The h-index does not account for highly citated papers, which has led to the development of alternative citation metrics to address this limitation (Kumar, 2019). Therefore, while the h-index is useful for identifying the principal authors publishing on seagrass on Atlantic Coast, it should not be viewed as a precise measure of a researcher’s influence in the field.

In terms of article citations, which serve as a measure of impact (Donthu et al., 2021), the most cited paper was that of Smale et al. (2019), with 878 citations, published in *Nature Climate Change*. This study explores the global impact of heatwaves on the ocean, specifically addressing their effects on seagrasses and the services they provide. Also, the research found a correlation between an increased number of heatwave days and a decrease in seagrass density and population health.

The second most cited article (875 citations) is by Reusch et al. (2005), published in *Proceedings of the National Academy of Science* (PNAS). This study examined genotypic diversity as a potential substitute for species diversity in coastal ecosystems with low richness, helping to buffer against extreme climatic events in the context of climate change. The authors manipulated the number of *Z. marina* clones in a meadow in the Baltic Sea and found that the genotypic complementarity of the more diverse meadow enhanced biomass production, plant density, and faunal abundance, even under near-lethal water temperatures (Reusch et al., 2005). Both articles focused on the theme of climate change, which is particularly relevant today as rising temperatures and other environmental changes impact organisms across all of earth’s biomes, including marine environments (Trégarout et al., 2024).

The third most cited article (870 citations) is by Mumby et al. (2004), published in Nature. This study evaluates how mangroves enhance the biomass of coral reef fish communities in the Caribbean, focusing on the fish species that depend on mangroves and have suffered local extinction due to deforestation. Notably, it also examines seagrasses, identifying them as critical nurserys grounds for fishes that move into mangroves before migrating to coral reefs. While both the first and second articles addressed seagrasses, they do so in the context of their importance and management, highlighting their value as nursery grounds.

**Figure 9:**
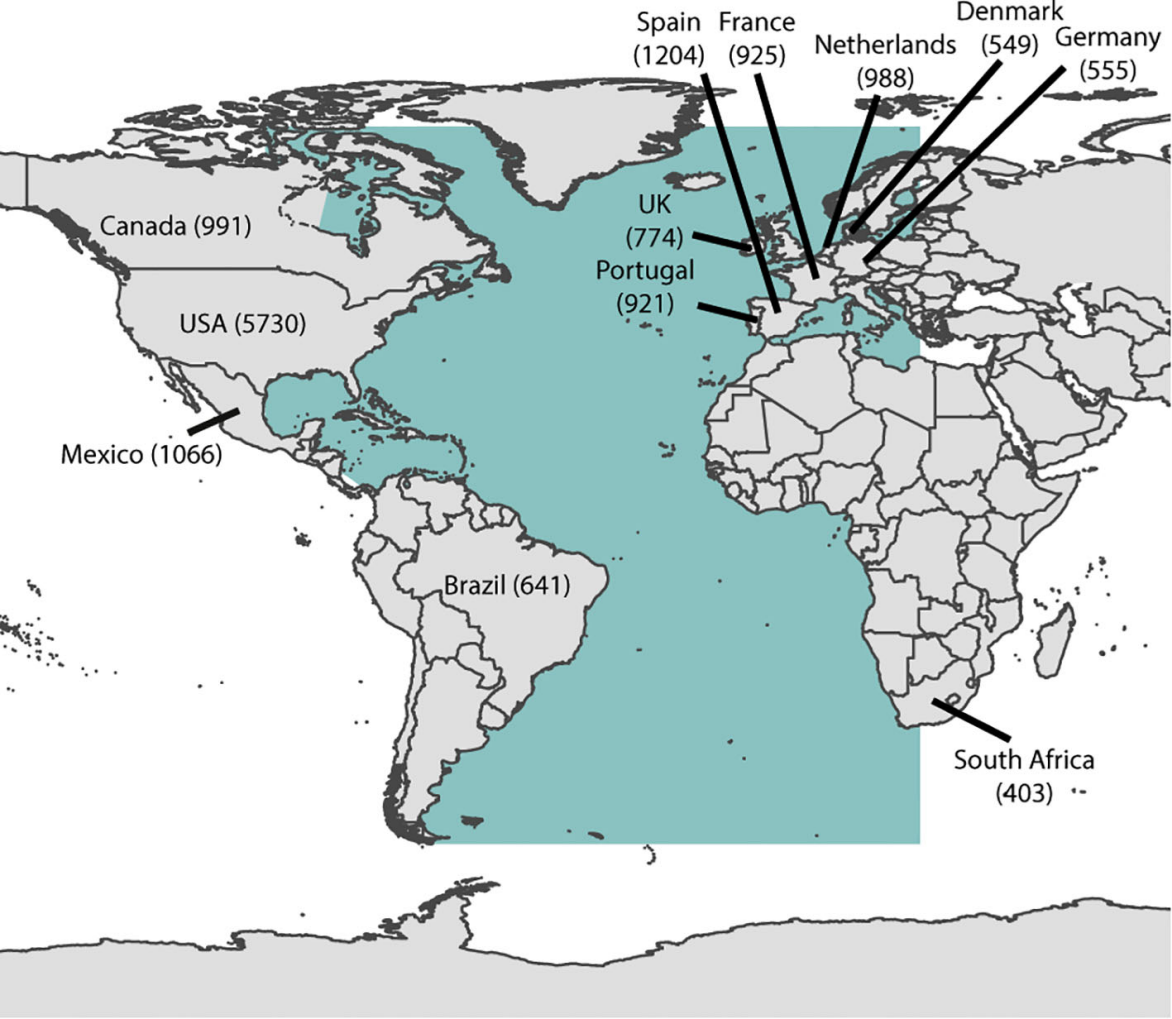
Total number of seagrass-related articles published by the most productive countries between 1969 and 2024.

The fourth most cited article was a comprehensive study on seagrass by Short et al. (2007), published in the Journal of Experimental Marine Biology and Ecology. This paper presents a bioregional model of seagrass distribution and diversity, establishing six bioregions for seagrass based on evolution history, ecology, and the coastal environments where seagrasses occur (estuaries, shallow coastal areas, lagoons). It also highlights the relationships with other flora and fauna (Short et al., 2007). Based on an analysis of the country of origin for all authors publishing on seagrass from the Atlantic Coast, the USA leads with (5.730 articles), followed by Spain (1.204), Mexico (1.066), Canada (991) and Netherland (988), France (925), Portugal (921), United Kingdon (774), Brazil (641), Germany (555), Denmark (549), South Africa (403) (**Figure 9**). This prominent position of the USA likely reflects the investments made by the National Science Foundation (NSF, 2024) through numerous grants since the 1970s, particularly those supporting the Seagrass Ecosystem Component.

The USA also had the highest number of corresponding authors (890), with 136 of these classified as multiple country publications (MCP), i.e. articles co-authored by researchers from different countries. Then, the collaborative nature of authorship across different countries explains why the number of publications in this analysis exceed the total considered in the review (3.440 articles), as one article may be counted multiple times. In addition to the USA, Portugal (154 authors, and 77 MCP) and Spain (195 authors 76 MCP) also showed significant contributions. The Universidad Nacional Autónoma de México published the highest number articles (177), followed by the University of Coimbra (160), the University of Florida (154), the Universidade of the Algarve (153) and the Florida International University (133). Although the USA has the highest number of articles and authors, the Universidad Nacional Autónoma de México (UNAM) is the leading institution in terms of publications. This is likely because American institutions are spread out across the Atlantic Coast, whereas Mexican authors are more concentrated at UNAM. A similar trend can be observed at the University of Coimbra.No African country stands out in seagrass research, except for South Africa, likely because West Africa is one of the least documented coastal regions regarding seagrass studies in the Atlantic Coast. However, a seagrass conservation project initiated in 2018 in West Africa has trained professionals to study and manage meadows of these marine plants. As a result more specific articles are expected for the region (Cheikh et al., 2023). One way to increase scientific production and knowledge in the Atlantic Coast is to invest in regions like South America and Africa, which that still have few studies. A recent scientometric study by Creed et al. (2023) on seagrasses in South America identified 314 documents, including grey literature, that mention seagrasses. Of these, 77 specifically addressed ecosystem services. This finding indicates that South America is aligning with broader trends in the Americas regarding research on ecology, climate change, and supporting services. The emphasis on seagrasses underscores their ecological significance and the growing recognition of their roles in providing essential services, such as carbon sequestration, habitat support, and water quality improvement. This trend reflects an increasing commitment to understanding and protecting these vital coastal ecosystems in the face of environmental challenges.

## 4 Concluding remarks

Recent reviews on seagrasses have emerged from various interests or objectives, highlighting the the significant global interest in these herbaceous phanerogams. Examples of some of these reviews have addressed issues such as status of pollutants and pollution in seagrass bed ecosystem (Zhang et al., 2023), the ecosystem services of seagrasses to identify knowledge gaps and to improve understanding of their roles (Lima et al., 2023), the economic value of these services (Dewsbury et al., 2016), and impact of climate changes (Tang and Hadibarata, 2022). However, most of these reviews are not specifically dedicated to the Atlantic Coast. One notable exception is a recent review that evaluates the ecosystem services provided by seagrasses on the southeast Atlantic coast of Brazil. This study examined areas and management forms of seagrass bioregions, as well as the impacts on these environments and their consequences for human well-being (Creed et al., 2023). Among the highlighted gaps revealed by our scientometric analysis was the low number of published articles from studies conducted in the South Atlantic compared to the North Atlantic, which has several relevant implications. The lower scientific production is a clear indicator of a presumably reduced level of knowledge about seagrasses in the South Atlantic. It is well known that the largest investments in research and development are made by developed countries in the North Atlantic, in contrast to those in developing countries. There are clear differences in overall public investment in education, science, technology and innovation (STI) between low- and middle-income regions and developed regions, which are also related to varying levels of human capital capacities in these areas (Okoye et al., 2022). In addition to these factors, equitable investment for inclusion is fundamental for achieving effective coastal zone management and the Sustainable Development Goals – ODS (Okoye et al., 2022). According to a report by the Organization for Economic Cooperation and Development (OECD), natural resources, climate changes and the environment are part of the megatrends influenced by investments in science, technology and innovation (ST&I), as well as human well-being (OECD, 2016). Thus, it is not difficult to envision that regular and planned investments in ST&I in South Atlantic countries could lead to a more equitable relationship between the development of seagrasses studies in the Coast and their natural consequences for preservation of regions where these organisms are vital.
